# The involvement of men in maternal health care: cross-sectional, pilot case studies from Maligita and Kibibi, Uganda

**DOI:** 10.1186/1742-4755-11-68

**Published:** 2014-09-05

**Authors:** Debra Singh, May Lample, Jaya Earnest

**Affiliations:** Kimanya-Ngeyo Foundation for Science and Education, Jinja, Uganda; International Health Programme, School of Nursing and Midwifery, Curtin University, Bentley, WA Australia

**Keywords:** Antenatal care, Male involvement, Maternal health outcomes, Uganda

## Abstract

**Background:**

The International Conference on Population Development held in Cairo in 1994 identified the importance of male involvement in reproductive health programs. Since then, there has been an increase in reproductive health initiatives that target both men and women in an attempt to fulfill the 5^th^ Millenium Development Goal. Yet, while the benefits of male involvement have been acknowledged, there continues to be a challenge in creating a space for and engaging men in maternal health. This is problematic due to the role of men as the head of the household in many countries, especially developing countries, which suffer from higher rates of maternal mortality. Furthermore, men are important as partners, fathers and health care professionals and as such it is important to involve and engage with men in maternal health education, and antenatal care.

**Methods:**

The purpose of this study undertaken in two rural villages in southeastern Uganda, was twofold: firstly to understand men’s current participation in antenatal, pregnancy care and childbirth and secondly to gain insight into both men and women’s attitudes toward increased male involvement. Focus group discussions and semi-structured questionnaires were used to collect information from 35 men and women. The women were either pregnant or had been involved in a birth experience in the past 3 years and the men had wives who were pregnant or had given birth recently.

**Results:**

Men interviewed in the two villages believed that issues related to pregnancy and childbirth were the domain of women. Involvement tended to be confined (to removed) strictly to traditional gender roles, with men’s main responsibility being provision of funds. The women, on the other hand, were interested in receiving more support from their husband through planning, attendance to antenatal care and physical presence in the vicinity of where the birth was taking place.

**Conclusion:**

This cross-sectional study has highlighted the space for increased male involvement and participation in maternal health, proposed recommendations and the need for community health education directed at men that engages them in this important area.

## Background

Considerable attention has been placed on maternal health outcomes as the 5^th^ Milleninum Goal. The 1994 International Conference on Population Development in Cairo was among the first international declarations of the importance of involvement of men in reproductive health programs. Men impact women’s reproductive health through their role as partners, fathers and healthcare workers. These effects can be direct and indirect, biological and social [1]. Since 1994, there has been an effort to increase the role of men in this area. This has mostly been done through the inclusion of men in family planning education. There continues to be a lack of emphasis on the involvement of men in maternal health care, yet they remain crucial partners in this. By developing projects that target men in their various roles in the community, maternal mortality will necessarily be impacted. It has been suggested that interventions should include “health education and community mobilization that aims to educate men on risk factors and danger signs associated with poor maternal health outcomes” [2].

Throughout sub-Saharan Africa, the area of pregnancy and childbirth is considered to be the responsibility of the woman. Therefore, it is rare to see men accompany women to antenatal care and be present for delivery [3]. Even in non-African settings men negotiating a space for their involvement can be challenging. Commenting on efforts in Europe to involve men in pregnancy and childbirth Plantin et al. [4] noted that many men felt marginalized and inadequately informed because most education focused on women with little space for their questions and concerns to be answered.

In Kenya, however a clear association was demonstrated between male attendance to at least one antenatal care visit and delivery by a skilled birth attendant [5]. In addition, there have been multiple studies that explore the space for men in the area of antenatal care, delivery and the postpartum period [6–11]. Specifically, men can encourage their wives to attend and accompany them to antenatal care, help prepare and save money for delivery, and arrange transportation to the birthing center, among other responsibilities [12]. In a 2010 study in the Gulu district of Uganda, researchers found that the outcomes associated with male attendance at antenatal care included knowledge of antenatal care services, women giving birth at a health clinic, distance to health facility and men desiring no further children. The study found that one of the strongest factors was knowledge about antenatal care services. Once men felt they knew more about antenatal care, they felt more drive to accompany their spouses [13].

Since the primary causes of maternal death are the result of the three delays; delay seeking care, delay reaching health care facilities and delay at an institution level in providing appropriate care [14] male involvement is critical. Delays that occur can often be the result of women seeking support from the head of the household, often men. This is especially the case when the situation involves the need for funds [2]. Involving men would allow them to support their partners to prepare for the delivery and seek out emergency care if necessary [3].

At the same time the involvement of men requires consideration regarding the effects of inclusion in issues previously considered the domain of women. There is also a question of whether men’s involvement will contribute to their perceived power over women [15]. Unequal structures or power dynamics within a community can have an impact on male involvement in women’s reproductive health [1]. Although there may be advantages to having men involved in maternal health, it is not clear if women would find this involvement acceptable and how it could be negotiated. It is important that patriarchal patterns are not repeated through the involvement of men and that women also want their partner’s assistance in pregnancy and delivery [10]. A 2001 study of condom use and decision making in Uganda demonstrated, however, that the effect of empowering women does not take away from the effects of empowering men, rather it enables both to make informed decisions [16].

Yet despite the documented benefits, in Uganda, male attendance at antenatal care is only around 10% [13]. Therefore, we wanted to study the barriers as well as the space for men’s participation in maternal care in Jinja, a city in the southeastern region of Uganda.

The main aims of the study were:

1. To explore the barriers, and spaces for, male involvement in Uganda in maternal healthcare;
2. To examine the current participation of men during pregnancy and delivery;
3. To describe women’s attitudes towards male involvement during pregnancy and delivery;
4. To explore the opportunity for openness, dialogue, more responsibility sharing during pregnancy and delivery.

## Methods

This study took place in Maligita and Kibibi, located in the southeastern region of Uganda in the Buganda and Busoga regions respectively. Both villages are located approximately 30 kilometers outside of Jinja, the closest major town. Jinja main hospital is the main referral centre where all women in the area go should a caesarean section or blood transfusion be required. The village of Maligita has a trained Ugandan midwife, supported by a faith-based organization that assists with antenatal care and supplies. Both Maligita and Kibibi have a Health Center IV, located 10 kilometers away as well as a closer Health Center II (see Table 1) approximately 2 km from each village. The villages were chosen because they would both face similar challenges in terms of distance from emergency care during labor, during which time engagement of men in transfer to hospital can be critical.

**Table 1:** **Hierarchy and roles of the health centre’s in Uganda**

Infrastructure level	Administrative level	Target population	Services provided
Health center I	Village	1,000	Community based health care prevention, Village Health teams
Health center II	Parish	5,000	Preventative, curative care, outreach
Health center III	Subcounty	20,000	Preventative, curative, outreach, inpatient, maternity, laboratory services
Health center IV	County	100,000	Preventative, curative, outreach, inpatient, blood transfusion and surgical care

This cross-sectional study incorporated the use of a simple questionnaire as well as focus group discussions (FGDs) for groups of men and women who had recently been involved in a birth experience. The questions for both the FGDs and the questionnaires were trialed with a private community based midwife and village health team member. Questions were centered on delivery, preparations, antenatal care, health services, involvement of men and factors impacting pregnancy and labor. Thirty-five individuals, 23 female and 12 male, completed questionnaires in Luganda or Lusoga (the local languages spoken in the area) - 19 from Maligita and 16 from Kibibi.

The five focus groups conducted were split between women who had recently given birth or were pregnant and men whose partners had recently given birth or were pregnant. In Maligita the women were separated into either currently pregnant women or those that had recently given birth, whereas in Kibibi the women combined into one group. Each group had between 5–12 participants. The FGDs were moderated by the second author with the assistance of a key informant, while recording and transcription were undertaken by the first author. The questionnaires were either self administered where the participant was sufficiently literate or with the assistance of a research assistant if needed. Questionnaires were completed prior to the FGDs.

Participants (see Tables 2 and 3) were recruited using purposive and opportunistic sampling through our key informants. In Maligita these were CM, a private community based midwife and a local Village Health Team member who helped recruit participants for the focus group, served as translators and assisted participants with filling in the questionnaire, where needed. In Kibibi, the key informants were a local Village Health Team member as well as an educational group coordinator. They helped organize focus group discussions and assisted with the translation and completion of questionnaires if needed.

**Table 2:** **Summary of participant characteristics**

	Maligita					
	Male		Female		Total	
	N	%	N	%	N	%
**Religion**						
Christian	4	67%	7	54%	11	58%
Muslim	2	33%	6	46%	8	42%
**Marital status**						
Single	0	0%	2	15%	2	11%
Married	6	100%	10	76%	16	84%
Divorced	0	0%	1	8%	1	5%
Widowed	0	0%	0	0%	0	0%
**Age**						
18-28	2	33%	8	62%	10	53%
29-39	1	17%	5	38%	6	32%
40-50	2	33%	0	0%	2	10%
Over 50	1	17%	0	0%	1	5%
**Number of children**						
1-3	2	33%	8	62%	10	53%
4-7	2	33%	4	30%	6	32%
8-11	2	33%	1	8%	3	16%
N/A	0	0%	0	0%	0	0%
**Education**						
Primary	2	33%	6	46%	8	42%
Secondary	4	67%	4	30%	8	26%
Post-secondary	0	0%	1	8%	1	5%
None	0	0%	2	15%	2	11%
**Education status**						
Studying	0	0%	0	0%	0	0%
Left school	6	100%	11	85%	17	88%
Never went	0	0%	2	15%	2	11%

**Table 3:** **Summary of participant characteristics**

	Kibibi					
	Male		Female		Total	
	N	%	N	%	N	%
**Religion**						
Christian	0	0%	4	40%	4	25%
Muslim	6	100%	6	60%	12	75%
**Marital status**						
Single	0	0%	1	10%	1	6%
Married	6	100%	9	90%	15	94%
Divorced	0	0%	0	0%	0	0%
Widowed	0	0%	0	0%	0	0%
**Age**						
18-28	0	0%	8	80%	8	50%
29-39	3	50%	2	20%	5	31%
40-50	3	50%	0	0%	3	19%
Over 50	0	0%	0	0%	0	0%
**Number of children**						
1-3	0	0%	4	40%	4	25%
4-7	4	67%	6	60%	10	63%
8-11	1	16%	0	0%	1	6%
N/A	1	16%	0	0%	1	6%
**Education**						
Primary	1	16%	6	60%	7	44%
Secondary	5	83%	4	30%	8	50%
Post-secondary	0	0%	0	0%	0	0%
None	0	0%	0	0%	0	0%
**Education status**						
Studying	0	0%	0	0%	0	0%
Left school	6	100%	10	100%	16	100%

Those included in the study were women currently pregnant or having given birth in the past 1–3 years or husbands of such women. Four slightly different questionnaire versions were administered to reflect the category they were in. The participants were provided with a snack and drink during the FDGs. All participants were over 18 years.

Our study was approved by the Human Research Ethics Committee at Curtin University in Western Australia. The study was discussed and permission sought from the local leader of the sub-county who supported the study. Each of the participants was given a participant information sheet in Luganda and provided informed consented via a signature or thumbprint. Participants were informed that they could withdraw from the study at any time if required. Participants were also ensured of anonymity and were assigned a pseudonym.

### Analysis

The data were transcribed during the focus groups with pseudonyms attached to each respondent. A multidisciplinary team then undertook thematic analysis. Instances of discussion about “antenatal care”, “preparation for birth”, “male involvement” and “male attendance” was checked for, and codes were created to reflect the interviewees’ statements. Emerging codes were systematically developed from the data. The researchers discussed emerging categories and themes. Recoding was done on the basis of group consensus. Standard descriptive statistics (frequencies and percentages) were used to summarize the responses to the demographic data and questionnaire questions which were of a closed-ended nature. Cross-tabulations of question responses against gender were performed to identify any differences between male and female respondents.

## Results and discussion

Of the twelve men who completed a questionnaire, all with the exception of one, believed they were either involved or very involved in the decision-making surrounding their wives’ pregnancy. A question was asked regarding whether it was common for men and women to make a decision together as to where delivery would take place. For the women of Kibibi, five said it was common while five said it was not common for the couple to decide together. In Maligita, nine of the women replied that it was common while three said it was not and one did not offer a clear answer. The majority of the men in Kibibi thought it was common for men and women to decide together, with five answering in the positive while one did not answer the question clearly. In Maligita, four men answered that it was common while two did not believe it was (see Table 4).

**Table 4:** **Is it common for men and women (the husband and wife) to decide where childbirth should take place?**

	Yes		No		N/A	
	Women	Men	Women	Men	Women	Men
Maligita	9	4	3	2	1	
Kibibi	5	5	5	0		1

In order to ascertain the reaction towards women making unilateral healthcare decisions, a question was asked if a women decided to go to the clinic or take her child to a clinic without discussing with her husband, would this cause a problem in the family? Of the ten women who completed the questionnaire in Kibibi, eight said yes it was a problem, with three specifically citing that their husbands would refuse to pay, while two explained that it would not be a problem. Of the 13 women who completed the questionnaire in Maligita, ten said it would be a problem with one specifically citing her husband’s unwillingness to pay while three said it was no problem. For the six men who completed the questionnaire in Kibibi, four said it was a problem for women to unilaterally seek care, while two said it was not. In Maligita, four said it was not a problem while two stated that it was problematic (see Table 5).

**Table 5:** **Would taking a child to a clinic without your husband’s permission cause a problem?**

	Yes		No	
	Women	Men	Women	Men
Maligita	10	4	3	2
Kibibi	8	2	3	4

With the exception of two men, all the men remarked that they were interested in attending antenatal care with their wives. This seemed to be an area in which women felt their husbands were the most absent. Several women related maternal mortality to a husband’s lack of interest in educating themselves to assist their wives by not attending antenatal care. Several of the men explained their reasoning for not attending antenatal care:

*“Most of the men here do not want to escort their women. Sometimes we do not have the means so we have to spend the whole day working to provide. If we have to work, we do not have time to escort our wives.” (Male participant from Kibibi men’s focus group)*

*“The problem is with our traditions, because a long time ago our fathers and our great great grandfathers did not escort their wives to antenatal care or even go to delivery. Since that is traditional, it is really hard to change that now.” (Male participant from Kibibi men’s focus group)*

In most instances it wasn’t that the men did not understand the importance of antenatal care, but rather that it just was not a common practice:

*“Most of the men, like they said, don't want to escort their women for antenatal care. But like many have said, you are supposed to because many things can happen.” (Male participant from Kibibi men’s focus group)*

Among both the men and women interviewed there was a sense that there are certain roles that men could fulfill in order to help their wives and acquiring money was key amongst them. All the women interviewed identified money as the key stumbling block to being able to prepare for the delivery to the best of their abilities:

*“Men should save some money. The husband has to save money to be used during that period because if there is any type of problem they are going to ask you for money. Because they greatest number of people who die during that period have no money.” (Female participant from Kibibi women’s focus group)*

*“Paramount is look for funds and be there. Because when she is pregnant she needs to eat well, she has to go for medical care, at least four times, and be aware of delivery” (Male participant from Maligita men’s focus group)*

Even though money was considered one of the key contributions for men, women continued to make efforts to save their own money through their own efforts kept separate from their husband. They referred to this as “woman’s money.”

In Kibibi, it was universally agreed upon that men did not attend delivery. None of the men of Kibibi marked attending the delivery as an area in which they wanted to participate more. With the exception of one woman, the women of Kibibi agreed that they did not want their husbands to be with them during delivery. Yet the reasons behind their belief differed for the men and women:

*“(Just over half) of the men take you to hospital and stand aside and wait. Those take care of you if there is a problem, they talk to the health workers, they really take care of you in the hospital. The others will stay in the village. When the labor pain starts they are scared to be near you. When they see you in pain, they feel bad, they feel sad and they don't want to be near you.” (Female participant from Kibibi women’s focus group)*

*“Most of the women did not want the men to be there during delivery. The women feel they are not polite, not showing love, they don't want to show that to the men.” (Male participant from Kibibi women’s focus group)*

*“He doesn't go, he doesn't need to go there because whenever you go there, you lose respect among your fellow men. You have to leave the woman to fight her own battle.” (Male participant from Kibibi men’s focus group)*

Whilst, there was a degree of openness to the idea that there is still more room for men to be involved in pregnancy and childbirth. In Maligita village, the women believed that the men could be more involved and felt they were reluctant to do so:

*“[We] would like men to be more involved in pregnancy and labor. For one woman, [she] called husband and he had to come because CM could not manage alone. Men should escort women to health center” (Female participant from Maligita women’s focus group)*

*“Men are making themselves busy, if they are busy then let them come with money. Men send women barehanded.” (Female participant from Maligita women’s focus group)*

There also seemed to be a general negative feeling towards family planning among men. The men and women expressed that it was common for many in their villages to not be interested in family planning or STI testing. Further, they explained that this stemmed from a fear of the results and a lack of desire to handle negative test results as well as misinformation on treatment and survival of individuals with HIV:

*“Most men don't want family planning, so the women do it alone. The husbands get annoyed when they do family planning on their own.” (Woman from Kibibi women’s focus group)*

*“A man will find another woman who gives birth” (Woman from Maligita women’s focus group)*

*“Sometimes the women decide that is it enough, but due to some complications with pills or injections, she has some bleeding, but she can't tell the husband because he doesn't know, they have to stop taking contraception but then they get pregnant again.” (Female participant Maligita women’s focus group)*

*“Family planning has its own problems, like if they go they get problems with their husbands. The woman aren't interested in sex when they have family planning so it causes problems” (Female participant Kibibi women’s focus group)*

*“[I am] interested in STI treatment but [my] faith doesn't encourage this idea of family planning. Because the faith talks about delivery and not family planning” (Male participant from Kibibi men’s focus group)*

*“…after testing and he has been told he is he is infected with HIV, the result is just trauma” (Male participant from Maligita men’s focus group)*

The men agreed with the women and felt there was room for sensitization in the community. All the men in Maligita believed there were ways in which men could be more of assistance including attending antenatal care, making sure transport was available if there was a problem and knowing more about what she should eat during pregnancy. Three of the men even showed an interest in being in the room during labor.

The results document that men were comfortable allowing women to be in charge of their pregnancy and delivery, even those who felt that men are the head of the household. They encouraged women to seek antenatal care, understanding that it was in the best interest of the mother and child, but never felt it necessary for them to attend or to be there during delivery. Rather it was the man’s role to make sure there was money available for the delivery and postnatal care. Some men also identified other roles including making sure the women were eating properly, ensuring their wives were not straining themselves physically and taking care of the children during labor. Most men felt they simply did not have the time to attend antenatal care. It appeared that men were, for the most part, unaware of the beneficial impact their presence might have.

However, it seemed that women were not as happy with this arrangement as the men. While a minority of the female participants expressed little desire to see men take on new roles, there appear to be unequal power balances in the distribution of responsibilities. It seemed the women felt that the responsibility of pregnancy and delivery is placed on them with little, other than monetary support, from their husbands – which was often absent or inadequate. They explained that men were not willing to attend antenatal visits; only some took them to clinic or health center during deliveries and were not as educated about pregnancy and delivery as women. These findings were similar to other studies in Africa, which indicate low male attendance (10-16%) at antenatal sessions [8].

The disparity between the expectations of men and women might serve to explain why men feel they are actively involved while women believe men’s involvement is lacking. For example, from a woman’s perspective her husband’s absence from antenatal care comes from an unwillingness to participate, whereas men feel they are participating in other avenues and don’t feel the need to attend. Yet when asked specifically about whether they were open to attending antenatal care the majority of the men who completed the questionnaire answered in the affirmative.

While a greater number of the participants did feel that men and women decided together where to give birth, there was little evidence from the FGDs that there was much communication enabling men and women to negotiate roles in pregnancy and delivery and that much was left to cultural norms. For Kinanee and Ezekiel-Hart [17], men’s participation requires a social and behavioral change on the part of men. This is the arena in which men and women seem to be conflicted in Kibibi and Maligita. Men are navigating within previously established gender roles while the women seem to want the men to move beyond those roles.

This was clear through attitudes to family planning. Both men and women expressed concerns regarding family planning but their reasons for doing so were different. Both felt that using family planning inhibited chances of getting pregnant at later times even once they stopped using family planning. Individuals identified birth defects, excessive bleeding, and problems getting pregnant. However, women seemed more concerned with trying to decrease family size so while they had problems with family planning they still understood its benefits. Men were skeptical because of the reasons mentioned above but they also were not often interested in curbing family size. Several men even identified the presence of many children as a sign of man’s masculinity and wealth. This desire on the part of men for more children is something the women recognize and feel pressured to deliver and there was an underlying fear of being replaced by new wives.

The study provided some rich data from a small group of participants in two villages however there are a few limitations that need to be mentioned. The sample was small and thus it may not be possible to generalize the findings, however the recommendations proposed may be used in other similar settings in Uganda and Africa. The time and funding constraints meant that the study could not be expanded to include more villages in the area. Although the authors had all lived in Uganda they did not speak the language and thus some information and nuances could have been lost in translation. In spite of these limitations every attempt was made to maintain rigor by using an audit trail so that the study could be replicated in another setting. Member checks were undertaken so that findings could be shared with the research assistant and the private community based. All authors discussed the analysis, findings and recommendations.

## Conclusion

Among the participants in both communities, there was an interest in discussing issues related to the roles of men and women in pregnancy and childbirth and a desire to have real results come from these conversations, not merely to be content with reflecting on the current situation. The men of Kibibi seemed particularly interested in sharing the situation in their community from their perspective. They spoke openly about their contributions to maternal healthcare, or in their lack thereof.

During the focus group discussions participants proposed the following recommendations:

– That there is a need for continuation of space, dialogue and opportunities created during focus group discussions should continue and develop into a partnership to tackle maternal health care.

– That there be increased emphasis and conversation about women and men as partners. These types of partnerships would allow men and women to freely consult and make choices together that achieve similar and fulfilling reproductive goals.

– That effort is made to involve and engage men in the area of maternal health care and reproductive health and a reconceptualization of gender roles and responsibilities to possibly reduce gender inequalities in the community.

It is not unusual for men to be hesitant to shift gender responsibilities [18]. However, there was an openness to move beyond current traditional roles among the men, more so in Maligita, where men agreed and felt they had a greater responsibility to contribute and support their wives and were interested in developing avenues through which to do so. This study has highlighted that there is a desire among the men and women interviewed for a greater participation in maternal health care. Women would like men to be more involved and men also have an interest in knowing more about pregnancy and childbirth. It may be a matter of providing more information to men so that they understand the proven benefits of attending antenatal care and supporting their partners in giving birth with a skilled birth attendant. Our study revealed that cultural norms can appear to create insurmountable obstacles for change, when in reality culture is fluid and allows for changes over time.

## Authors’ information

Dr Debra Singh is a Medical doctor living in Jinja, Uganda and is the National Research and Collaboration Coordinator for the Kimanya-Ngeyo Foundation for Science and Education in Jinja and is a PhD candidate at University of Sydney. Jaya Earnest is currently the Director of Graduate Studies in the Faculty of Health Sciences and Associate Professor of International Health in the Centre for International Health at Curtin University in Western Australia. May Lample is currently a Public Health graduate student at UC Berkeley, majoring in maternal and child health.
